# Adipose Tissue from Lean and Obese Mice Induces a Mesenchymal to Epithelial Transition-Like Effect in Triple Negative Breast Cancers Cells Grown in 3-Dimensional Culture

**DOI:** 10.3390/ijms21176439

**Published:** 2020-09-03

**Authors:** Emmanuel C. Asante, Nikitha K. Pallegar, Alica J. Hoffmann, Alicia M. Viloria-Petit, Sherri L. Christian

**Affiliations:** 1 Department of Biochemistry, Memorial University of Newfoundland, St. John’s, NL A1B 3X9, Canada; easante@mun.ca (E.C.A.); nk2153@mun.ca (N.K.P.); ajhoffmann@mun.ca (A.J.H.); 2 Department of Biomedical Sciences, Ontario Veterinary College, University of Guelph, Guelph, ON N1G 2W1, Canada; aviloria@uoguelph.ca

**Keywords:** MET, metastasis, breast cancer, triple negative breast cancer, obesity, adipose tissue, 3D culture

## Abstract

Breast cancer is the second leading cause of cancer-related mortality among women globally with obesity being one risk factor. Obese breast cancer patients have at least a 30% increased risk of death from breast cancer compared to non-obese breast cancer patients because they present with larger tumors and generally have increased rates of metastasis. Moreover, obese breast cancer patients respond more poorly to treatment compared to non-obese patients, particularly pre-menopausal women diagnosed with triple negative breast cancer (TNBC). To help understand the molecular mechanisms underlying the increased metastasis associated with obesity, we previously established a three-dimensional culture system that permits the co-culture of adipocytes and TNBC cells in a manner that mimics an in vivo milieu. Using this system, we demonstrate that white adipose tissue from both lean and obese mice can induce a partial mesenchymal-to-epithelial transition (MET). Triple negative breast cancer cells adopt an epithelial morphology and have an increased expression of some epithelial markers, but they maintain the expression of mesenchymal markers, furnishing the breast cancer cells with hybrid properties that are associated with more aggressive tumors. Thus, these data suggest that adipose tissue has the potential to promote secondary tumor formation in lean and obese women. Further work is needed to determine if targeting the partial MET induced by adipose tissue could reduce metastasis.

## 1. Introduction

Breast cancer (BC) is one of the most prevalent and deadliest cancers among women [1]. It is ranked second to lung cancer as the leading cause of all cancer deaths [2]. An independent risk factor for BC is obesity [3]. Women who are obese have a 30% increased risk of death from BC compared to non-obese women due to larger tumor sizes and increased rates of metastasis [1,3,4,5]. Post-menopausal obese women are at an increased risk of death from estrogen receptor or progesterone receptor-positive BC, while obese pre-menopausal women are most likely to die from triple negative breast cancer (TNBC) [6]. Furthermore, obese BC patients respond more poorly to treatment compared to non-obese patients, especially those with TNBC [6,7]. The reasons behind the poor treatment outcome are not fully understood; however, obesity is associated with the systemic elevation of insulin-like growth factor 1 (IGF-1), adipokines, cytokines, and pro-angiogenic factors, which create a pro-oncogenic environment [8].

The primary cause of morbidity and death among BC patient is tumor metastasis [9]. The most common sites of BC metastasis include the bone, brain, lungs, and liver [10]; although rare, the tumor can also spread to the extraocular muscle [11] and to the urinogenital regions of the body [12,13,14]. Tumor metastasis is a highly complex and multistep event. First, tumor cells dissociate from the primary tumor mass and may undergo an epithelial-to-mesenchymal transition (EMT). EMT endows tumor cells with invasive and migratory properties [15]. This is often followed by a period of increased proliferation and then by a mesenchymal-to-epithelial transition (MET) that allows tumor cells to colonize secondary sites [15]. Evidence shows that cancer cells can successfully metastasize to other organs without completely undergoing EMT [16,17]. The metastasizing tumor cells may exhibit a state of phenotypic duality where tumor cells possess both mesenchymal and epithelial properties [18,19,20]. Tumor cells in this hybrid mesenchymal/epithelial (M/E) state have migratory and cell-cell adhesion properties and tend to migrate as multicellular aggregates [18,21,22]. In addition, these tumor cells have superior survival advantages than cells that had undergone complete EMT and are migrating as single cells [18,23]. For instance, cells migrating in clusters are more likely to survive anoikis en route to distal organs [24,25]. Furthermore, they have been shown to be immunoresistant and chemoresistant [23]. Thus, a hybrid tumor state can contribute to increased tumor aggression.

Adipocytes can induce the expression of mesenchymal markers and promote the invasion and migration of BC cells as well as tumor cell proliferation via the secretion of adipokines (e.g., leptin), and hormones (e.g., estrogen) [26,27]. Moreover, obesity has been linked to visceral metastases [14] where adipocytes from visceral white adipose tissue (WAT) have enhanced effects on the EMT of BC cells compared to those from subcutaneous WAT [15]. This effect may be related to the observation that adipocytes from visceral and subcutaneous WAT have different metabolic and gene expression profiles, different adipokine secretion profiles, and different diet-induced responses [16]. Furthermore, in the obese state, WAT suffers system-wide chronic inflammation secondary to the excessive deposition of lipids and expansion of the adipocyte [28]. This results in the activation of the nuclear factor kappa B (NF-κB) pathway and elevated secretion of leptin, as well as pro-inflammatory cytokines such as IL-1β, IL-6, and IL-8, tumor necrosis factor alpha (TNF-α) and vascular endothelial growth factor (VEGF) [29]. Elevated levels of pro-inflammatory cytokines are strongly associated with increased cancer risk and tumor progression [30].

Our previous study demonstrated that mature adipocytes can induce a MET-like change in the mesenchymal MDA-MB-231 and HS478t TNBC cell lines when in three-dimensional (3D) culture and in the presence of a laminin-rich extracellular matrix, but they have no effect on SUM159 (mixed morphology) or MCF7 (epithelial) cells [31]. Evidence shows that this process can be at least partially mediated by factors secreted by adipocytes. Therefore, this suggests that adipocytes may play a role in MET-mediated secondary tumor establishment. Taken together, it can be surmised that the key to elucidating the mechanisms underlying obesity-induced tumor aggression and metastasis lies in understanding how adipocytes, especially in the obese state, interact with and affect tumor cells. To that end, we utilized 3D culture and organotypic models to assess the effects of lean and obese mouse WAT on the MET of the mesenchymal MDA-MB-231 TNBC cell line. Our findings show that conditioned media (CM) from both lean and obese mice WAT can induce a MET-like change in mesenchymal MDA-MB-231 TNBC cell line. Interestingly, the degree of WAT-induced MET-like changes in TNBC cells was adipose depot-dependent. Our findings shed light on a previously undescribed role of the adipose tissue in promoting a hybrid M/E state that may enhance secondary tumor formation.

## 2. Results

### 2.1. Lean and Obese White Adipose Tissue Causes Mesenchymal MDA-MB-231 TNBC Cells Grown in 3D Culture to Acquire an Epithelial-Like Morphology

**Figure 1:**
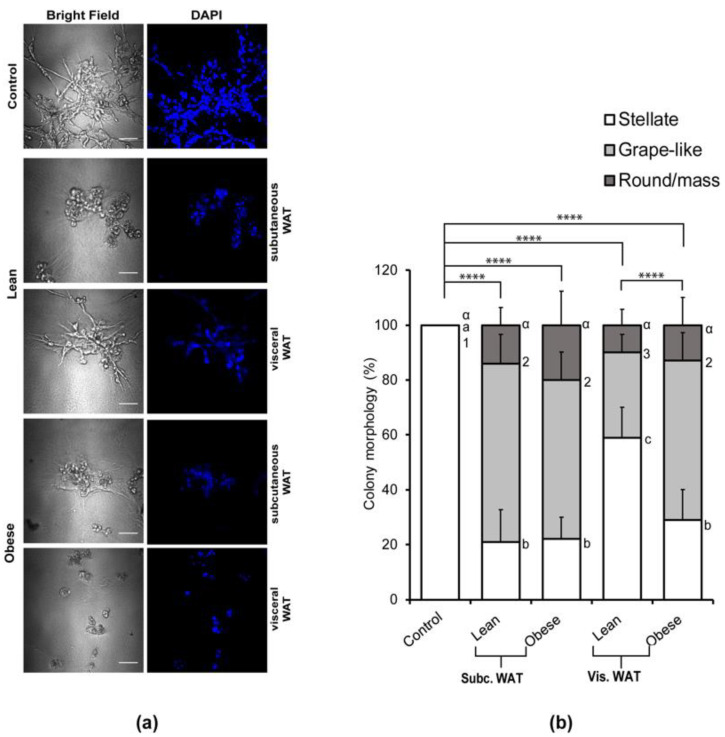
White adipose tissue (WAT) conditioned media alters the characteristic morphology of mesenchymal triple negative breast cancer cell line. (**a**) Representative images of MDA-MB-231 cells grown in a 3D culture with or without lean or obese subcutaneous or visceral adipose tissue conditioned media (CM); scale bar = 100 µM. (**b**) Total percentages of structure shapes are shown as mean ± SD (n = 6 biological replicates). The overall significance of the proportion of colony shapes between the groups was assessed by χ^2^ analysis, **** *p* < 0.0001. Significant differences between groups were determined by one-way ANOVA followed by Tukey’s HSD post hoc analysis. Different letters or symbols represent statistically different groups, ^a,b,c^ *p* < 0.05 for stellate, ^1,2,3^ *p* < 0.05 for grape-like, and ^α^ *p* < 0.05 for round/mass-like.

To assess the effect of mouse WAT on the colony morphology of mesenchymal TNBC cells grown in 3D, 400 mg of peri-uterine (visceral) and inguinal (subcutaneous) WAT were isolated from lean mice (21.4 ± 1.0 g at 16 weeks old) and obese mice (28.1 ± 2.2 g at 16 weeks old) (Figure S1). Then, these depots were cultured ex vivo to generate white adipose tissue-conditioned media (WAT-CM). As expected, MDA-MB-231 cells grown in 3D culture without WAT-CM maintained their characteristic stellate morphology (Figure 1a). In contrast, cells grown with WAT-CM acquired a mixed population of stellate, grape-like, and round/mass-like colony morphologies in a manner that was dependent on both depot and obesity (Figure 1a). Of note, mesenchymal BC cells adopt a stellate morphology in 3D culture, while epithelial BC cells have a grape-like or round/mass-like morphology [32]. The lean and obese mouse subcutaneous WAT had similar effects on the morphological alteration of MDA-MB-231 cells in 3D culture. Both caused a significant decrease in the proportion of stellate colony in comparison to control, with 60% and 20% of the cell colonies acquiring grape-like and round/mass-like morphologies, respectively. The lean mouse visceral WAT had the least effect on the colony morphology of the MDA-MB-231 cells with 60% of the colonies retaining their characteristic stellate morphology and only 30% and 10% of the colonies acquiring grape-like and round/mass-like morphologies, respectively. The obese mouse visceral WAT, on the other hand, had similar effects as the lean and obese mouse subcutaneous WAT with 29% of MDA-MB-231 cells retaining their stellate colony morphology, while 58% and 13% of the colonies had acquired grape-like and round-like morphologies, respectively.

While both lean and obese mouse subcutaneous and visceral WAT could cause mesenchymal MDA-MB-231 TNBC cells to acquire an epithelial-like morphology in 3D culture, the difference actually lies in the adipose depot. Overall, the subcutaneous WATs (both lean and obese) were most potent in inducing the morphology changes, but the visceral WAT could only induce significant morphology changes in the obese state.

### 2.2. Lean and Obese White Adipose Tissue Induces Epithelial Biomarker Expression and Maintenance of Mesenchymal Biomarker Expression

**Figure 2:**
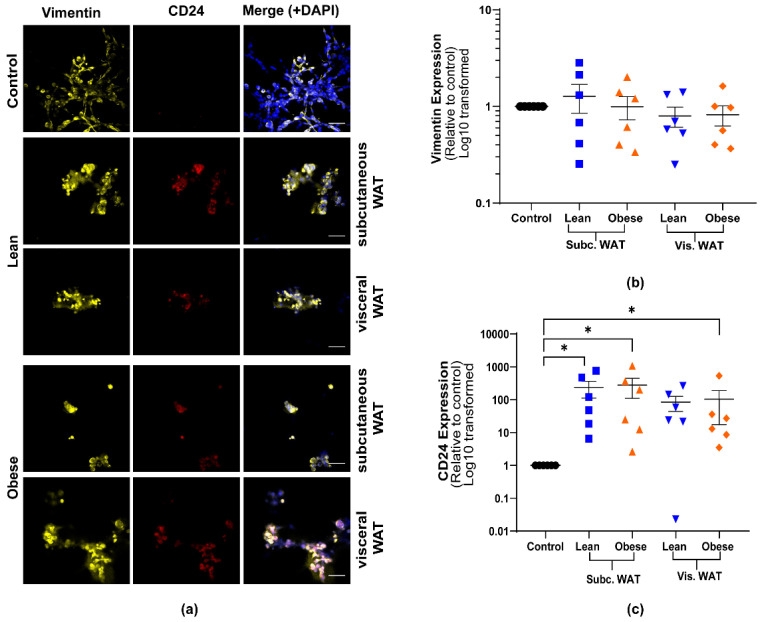
Mouse WAT-CM induces the expression of CD24 in mesenchymal MDA-MB-231 cells but does not affect the expression of vimentin. Representative images of MDA-MB-231 cells cultured with or without lean or obese subcutaneous or visceral WAT-CM and co-stained for vimentin, CD24, and DAPI (**a**); scale bar = 100 µM. (**b**) and (**c**) are the relative expression of the respective markers, mean ± SEM (*n* = 6 biological replicates). The average protein expression from five images per replicate were normalized to DAPI and analyzed relative to the control cultures. Significance was determined by Friedman’s test. If significant, Dunn’s test was used for further pairwise comparison; * *p* < 0.05.

**Figure 3:**
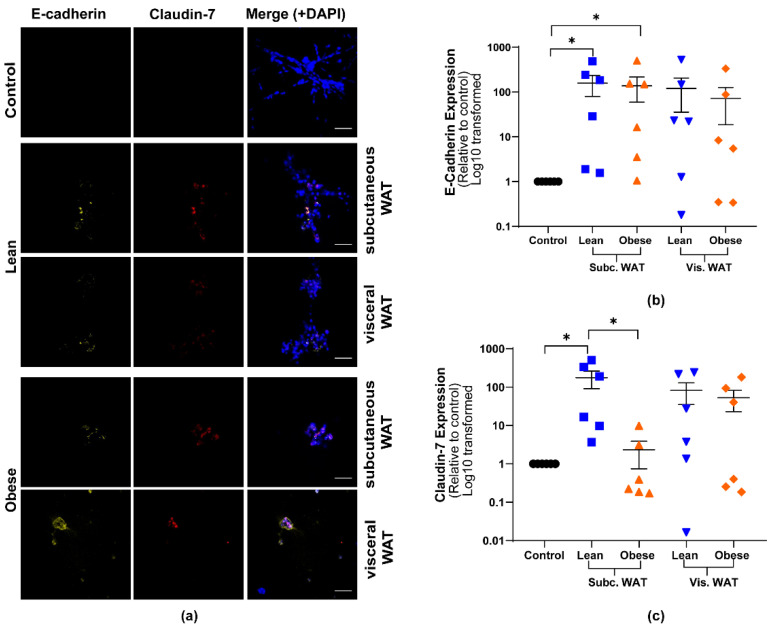
Mouse WAT-CM induces the expression of epithelial biomarkers in mesenchymal triple negative breast cancer cell line grown in a 3D culture. (**a**) Representative images of MDA-MB-231 cells cultured with or without lean or obese subcutaneous or visceral WAT-CM and co-stained for E-cadherin, claudin-7, and DAPI; scale bar = 100 µm. (**b**) and (**c**) are the relative expression of the respective markers, mean ± SEM (n = 6 biological replicates). The average protein expression from five images per replicate were normalized to DAPI and analyzed relative to the control cultures. Significance was determined by the Friedman test. If significant, Dunn’s test was used for further pairwise comparison; * *p* < 0.05.

Having observed that mouse WAT-CM can cause mesenchymal MDA-MB-231 cells to acquire rounded colony morphologies in 3D culture, we next assessed the effect of WAT-CM on the expression of EMT biomarkers in order to determine if the morphological changes reflected an MET event (Figure 2 and Figure 3). Immunofluorescence (IF) confocal microscopy was used to detect the expression of EMT protein markers in MDA-MB-231 TNBC cells cultured in 3D with or without mouse WAT-CM. The expression of vimentin (a mesenchymal marker), E-cadherin and claudin-7 (epithelial markers), and CD24 (a stemness and an epithelial maker) were assessed [33].

As expected, MDA-MB-231 cells cultured in 3D without WAT-CM expressed high levels of vimentin but had low or no expression of E-cadherin, claudin-7, or CD24 (Figure 2 and Figure 3). WAT-CM from lean and obese mice induced the expression of CD24, claudin-7, and E-cadherin in MDA-MB-231 cells relative to control but had no effect on the expression of vimentin. There was no statistically significant difference in the expression of the individual biomarkers between lean and obese WAT-CM (Figure 2 and Figure 3). Furthermore, while lean and obese mouse WAT-CM induced the expression of claudin-7 in MDA-MB-231 cells, this increase was lower in cells treated with obese mouse subcutaneous WAT-CM (Figure 3c). Overall, these data show that both lean and obese mouse WAT-CM can induce the expression of epithelial biomarkers in mesenchymal MDA-MB-231 cells.

### 2.3. MET-Like Changes Induced by WAT-CM from Obese Mice in MDA-MB-231 Cells Is Not Dependent on the Quantity of Adipose Tissue

One of the phenotypic hallmarks of obesity is the overabundance of adipose tissue [34]. Previous studies have established that obesity favors tumor metastasis relative to the non-obese state [35,36,37]. Obesity can promote tumor metastasis via different mechanisms such as increased induction of EMT in tumor cells [38]; therefore, WAT-induced MET-like changes in 3D cultured MDA-MB-231 cells could be dependent on a qualitative change to WAT in the obese state as well as the quantity of WAT present. Thus, we hypothesized that the greater the quantity of obese adipose tissue present, the stronger the MET-like effect. To test this hypothesis, MDA-MB-231 cells grown in 3D were treated with or without WAT-CM generated from an ex vivo culture of 200 mg or 800 mg of obese mouse subcutaneous or visceral WAT. We used obese WAT for this experiment because only approximately 500 mg of WAT can be isolated from the subcutaneous or visceral depots of a lean mouse; therefore, WATs would have to be pooled from multiple lean mice to obtain enough quantity for the experiment, which negatively impacts our ability to capture biological variability.

**Figure 4:**
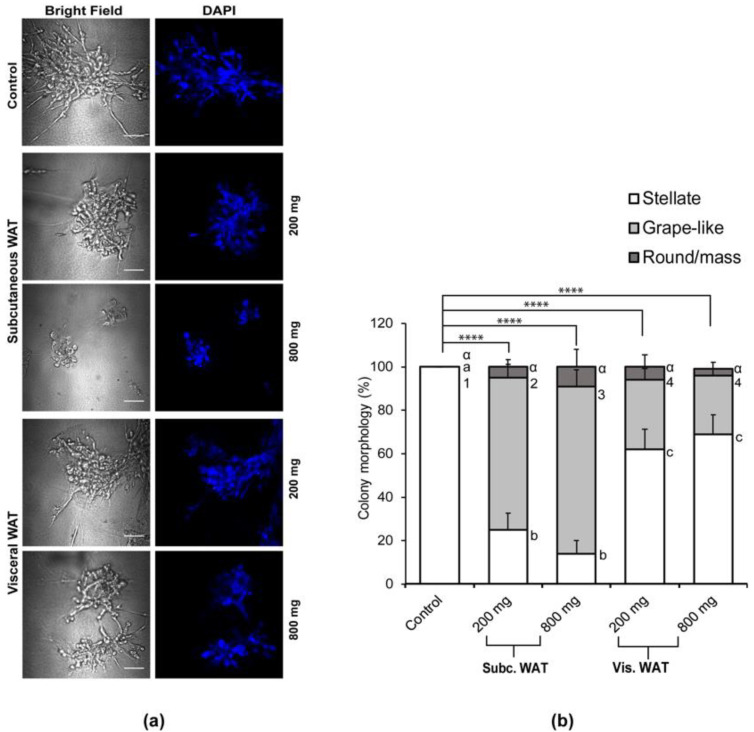
Effect of varied WAT masses from obese mice on colony morphology changes of MDA-MB-231 cells in 3D culture. (**a**) Representative images of MDA-MB-231 cells grown in a 3D culture with or without 200 mg or 800 mg of obese moue subcutaneous or visceral adipose tissue conditioned media (CM); scale bar = 100 µM. (**b**) Total percentages of structure shapes are shown as mean ± SD and were computed from six biological replicates. Overall significance of the proportion of colony shapes between the groups was assessed by χ^2^ analysis, **** *p* < 0.0001. Significant differences between groups were determined by one-way ANOVA followed by Tukey’s HSD post hoc analysis. Different letters or symbols represent statistically different groups, ^a,b,c^ *p* < 0.05 for stellate, ^1,2,3^ *p* < 0.05 for grape-like, and ^α^ *p* > 0.05 for round/mass-like.

Similar to our previous observation, the morphological analysis of the cell colonies formed post-WAT-CM treatment revealed a mixed proportion of different colony morphologies (Figure 4a). Overall, there was a significant decrease in the proportion of stellate cell colonies and an increase in the proportion of grape-like colonies. For subcutaneous WAT-CM, cells in the 800 mg treatment group formed more grape-like colonies than those in the 200 mg treatment group. However, there was no significant difference in the proportions of colony morphologies between cells treated with 200 mg or 800 mg of the obese visceral WAT-CM (Figure 4). Of note, cells treated with either amount of subcutaneous WAT formed significantly lower proportions of stellate colonies and a higher proportion of grape-like colonies in comparison to cells treated with visceral WAT. This suggests that the subcutaneous WAT is more effective than the visceral WAT in altering the characteristic morphology of MDA-MB-231 cells grown in 3D, unlike previous data which showed that the visceral WAT in the obese state was as effective as the subcutaneous WAT.

**Figure 5:**
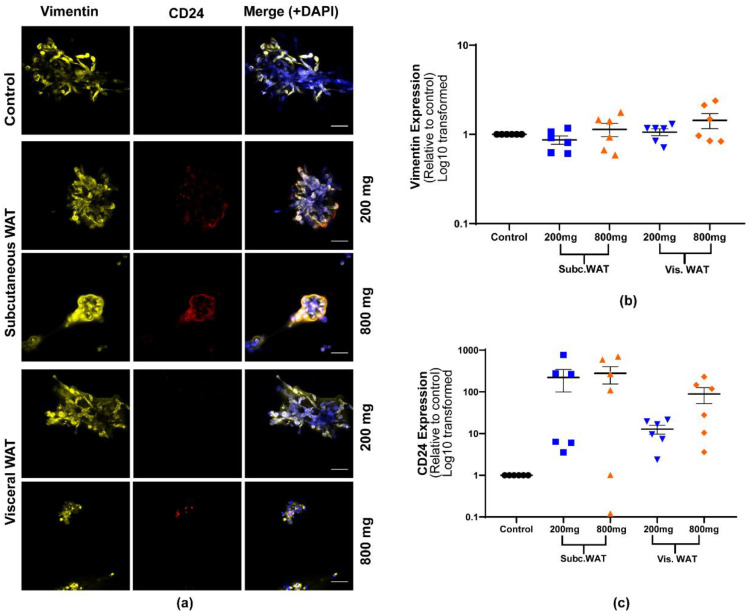
Effect of varied WAT masses from obese mice on vimentin and CD24 protein expression in MDA-MB-231 cells. (**a**) Representative images of MDA-MB-231 cells cultured with or without 200 mg or 800 mg of obese subcutaneous or visceral WAT-CM and co-stained with anti-vimentin, anti-CD24, and DAPI; scale bar = 100 µm. (**b**) and (**c**) are the relative expression of the respective markers, mean ± SEM (n = 6 biological replicates). The average protein expression from five images per replicate were normalized to DAPI and analyzed relative to the control cultures. No groups were found to be significantly different by the Friedman test.

**Figure 6:**
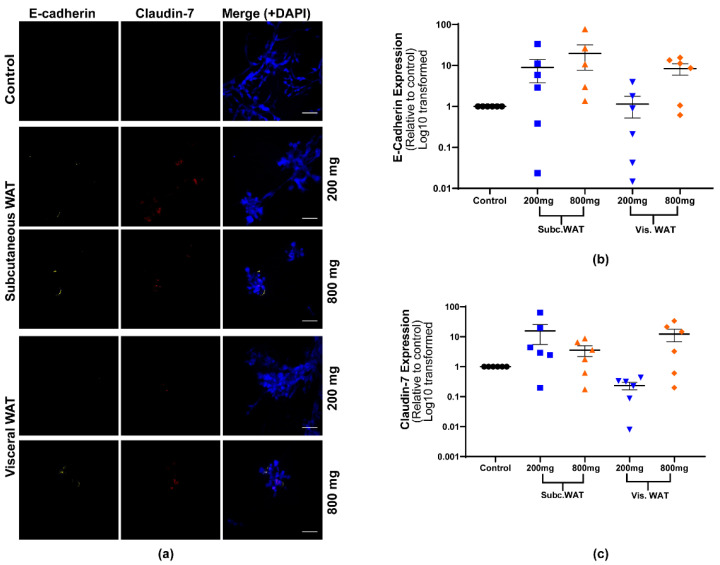
Effect of varied WAT masses from obese mice on epithelial biomarker expression in MDA-MB-231 cells. (**a**) Representative images of MDA-MB-231 cells cultured with or without 200 mg or 800 mg of obese subcutaneous or visceral WAT-CM and co-stained with anti-E-cadherin, anti-claudin-7, and DAPI; scale bar = 100 µm. (**b**) and (**c**) are the relative expression of the respective markers, mean ± SEM (n = 6 biological replicates). The average protein expression from five images per replicate were normalized to DAPI and analyzed relative to the control cultures. No groups were found to be significantly different by the Friedman test.

An analysis of the EMT protein marker expression showed that both 200 mg and 800 mg of subcutaneous and visceral WAT-CM can induce the expression of epithelial proteins in 3D cultured MDA-MB-231 cells with no effects on the expression of vimentin (Figure 5 and Figure 6). While the expression of all the epithelial biomarkers was generally elevated in all the treatment groups, it was consistently lower in cells treated with 200 mg of visceral WAT-CM (Figure 5 and Figure 6). Overall, there was no significant difference in the expression of the EMT biomarkers among cells cultured with 200 mg or 800 mg of subcutaneous or visceral WAT-CM. Taken together, the data show that the MET-like changes induced by obese WAT in 3D cultured MDA-MB-231 cells are not dependent on the quantity of WAT.

### 2.4. Assessment of Hybrid Mesenchymal/Epithelial Phenotype Status of WAT-CM Treated MDA-MB-231 Cells

**Figure 7:**
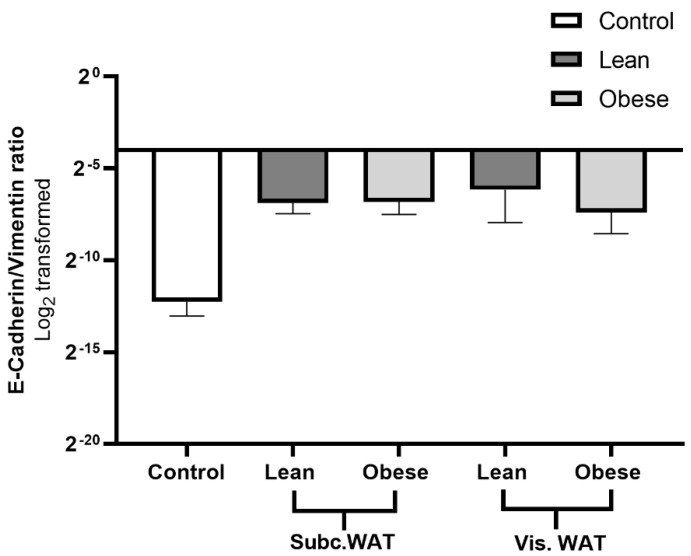
Log_2_-transformed ratios of E-cadherin and vimentin expression demonstrate a hybrid phenotype in WAT-CM-treated MAD-MB-231 cells. Log_2_-transformed E-cadherin/vimentin ratio was determined using the immunofluorescence data of MDA-MB-231 cells treated with or without WAT-CM in 3D culture. Cells that have stellate colony morphology and an E-cad/vim ratio < 0 are considered mesenchymal. Cells with grape-like/mass-like colony morphology and an E-cadherin/vimentin ratio > 0 are considered epithelial. Cells with mixed mesenchymal and epithelial colony morphologies and a discordant E-cadherin/vimentin ratio are considered as hybrid.

As observed in Figure 1, Figure 2, Figure 3, Figure 4, Figure 5 and Figure 6, MDA-MB-231 cells treated with WAT-CM in 3D culture presented with features that were suggestive of a hybrid M/E phenotype. The expression of vimentin in MDA-MB-231 cells remained elevated even upon acquiring epithelial morphology and enhancing epithelial marker expression. To quantitively assess the M/E status of WAT-CM treated cells, we calculated the Log_2_-transformed E-cadherin/vimentin (E-cad/vim) expression ratio of the cells and stratified cell populations into epithelial, mesenchymal, and hybrid phenotypes, as previously described [39,40,41]. Treatment groups with a Log_2_-transformed E-cad/vim ratio < 0 and a stellate colony morphology were considered mesenchymal. Groups with a Log_2_-transformed E-cad/vim ratio > 0 with a grape-like/mass-like colony morphology were classified as epithelial. Meanwhile, treatment groups with mixed colony morphologies of stellate and grape-like/mass-like structures and a discordant E-cad/vim ratio were considered hybrid in phenotype. The E-cad/vim ratios of the control groups, which are stellate in morphology (Figure 1), were less than 0; hence, they are considered mesenchymal in phenotype. On the other hand, MDA-MB-231 cells treated with WAT-CM had E-cad/vim ratios that were less than 0, even though these cells had a mixed colony morphology (Figure 7). This discordance in the colony morphology and E-cad/vim ratio corroborates the hybrid phenotype of the cells.

## 3. Discussion

The 3D culture model utilized in our previous study was designed such that differentiated 3T3-L1 adipocytes and breast cancer cells sandwiched a basement membrane matrix [31]. The basement membrane provides a physical medium through which the two different cells interact with each other. Although properties of mature adipocytes are preserved in in vitro differentiated adipocytes, obesity-associated characteristics such as increased pro-inflammatory cytokine secretion and macrophage infiltration are lost during the isolation, and the subsequent induced differentiation of primary adipocytes is never experienced by cell lines [42,43]. This led to the adaptation of an organotypic culture model in which mouse WAT was cultured ex vivo to generate WAT-CM. Then, TNBC cells grown in 3D on a basement membrane were treated with WAT-CM. This model permitted an assessment of the effects of secreted factors from the WAT on the TNBC cells via the CM.

Using this modified approach, we found that both lean and obese mouse subcutaneous and visceral WATs could cause MET-like changes in mesenchymal MDA-MB-231 cells, as evidenced by their acquisition of epithelial morphology and protein expression. In general, no marked difference was observed in the MET induction capacity of lean and obese mouse WAT; however, the subcutaneous WAT and visceral WAT affected MET-like morphology changes in MDA-MB-231 cells distinctively. Both lean and obese mouse subcutaneous WAT proportionately increased the “epithelialization” of MDA-MB-231 cells relative to the lean visceral WAT. Meanwhile, in the obese state, mouse visceral WAT induced the same magnitude of effect as the lean and obese mouse subcutaneous WAT. However, we observed quite a lot of biological variability within the groups, which may be due to differences between individual WAT cultures in terms of the particular cellular makeup of the depot sampled or due to the innate variability between mice. This variability may have masked any subtle effects in addition to reducing the statistical confidence in the differences observed. Subcutaneous and visceral WATs are morphologically and functionally distinct [44,45]. They arise from different progenitor cells and have a differential gene expression pattern [46,47]. In addition, the two depots also have distinct biomolecule and adipokine secretion profiles [44]. Therefore, the depot-dependent MET-like morphological changes may be due to the differential secretion of pro-MET factor(s) by the two depots, hence reflecting the fundamental biological differences between subcutaneous and visceral WAT.

Since the obese WAT has larger but fewer adipocytes than the lean WAT, we assessed the likelihood of a quantity-dependent MET-like effect by exposing MDA-MB-231 cells to CM from 200 mg or 800 mg of obese WAT. We found no appreciable difference in the MET induction capacity of the varied adipose masses, thus suggesting that a WAT-induced MET-like effect may not be dependent on the quantity of fat present. It is possible that at any given time, WAT secretes a sufficient amount of the unknown pro-MET factor that may be necessary to surpass the MET activation threshold where additional exposure does not have any further effects. Alternatively, there may be a negative feedback loop that limits the amount of this factor in a given incubation period. More work is needed to understand the absence of a dose response.

In principle, true MET is characterized by the following: a complete acquisition of epithelial morphology, downregulation of mesenchymal proteins, and upregulation of epithelial proteins [48,49]. In this study, we found that even though the WAT moderately upregulated the expression of epithelial biomarkers in MDA-MB-231 cells, the expression of vimentin, a mesenchymal biomarker, remained steady. Thus, MDA-MB-231 cells appeared to be in a hybrid M/E state. While we used fluorescence intensity to quantify these changes, confirmation with a more quantitative method, such as immunoblot, will be needed to fully validate this hybrid state. These observed changes in biomarker expression was not in keeping with our previous study, which showed that mature adipocytes caused a significant downregulation of the expression of vimentin, while it upregulated the expression of epithelial biomarkers [31]. Of note, in that study, mature adipocytes were co-cultured with MDA-MB-231 cells in 3D for 5 days. This differs from the CM approach we adopted, which exposed MDA-MB-231 cells to fresh CM twice within 5 days. It is possible that a constant and uninterrupted supply of the pro-MET factor(s) in the culture system is crucial for initiating enough signals to downregulate the expression of the mesenchymal biomarker, which is lacking in a CM approach. Furthermore, WAT is composed of different cell types, including neutrophils, lymphocytes, macrophages, endothelial cells, and stem cells [50], which may modify the effect of the adipocytes. The impact of the non-adipocyte fraction of WAT cells on the expression of M/E phenotype in MDA-MB-231 cells remains unknown. Furthermore, while this study did not assess the effect of non-adipose mouse tissues on MET of MDA-MB-231 cells, our previous study found that pre-adipocytes did not induce MET, suggesting that the effect was specific to mature adipocytes [31]. A more comprehensive analysis of the effect of different tissues on mesenchymal cancer cells will be needed to confirm if this truly an adipocyte specific effect.

While the clinical implications of a WAT-induced M/E hybrid phenotype in TNBC cells is currently unknown, it is possible that WAT-CM treated MDA-MB-231 cells were on course to a full MET. Gunasinghe and colleagues used an MDA-MB-468 xenograft model to demonstrate that BC cells undergoing local lymphovascular invasion could transition from a hybrid state to an epithelial state [49]. They showed that tumor emboli in the lymphovasculature which co-expressed vimentin and E-cadherin gradually lost the expression of vimentin to predominantly express E-cadherin. This evidence supports the idea that the hybrid phenotype observed in our study could be a transitionary phase which might be followed by the downregulation of vimentin possibly upon the amplification of a MET activation signal.

Although accumulating evidence indicates that the MET process plays a critical role in secondary tumor establishment, the underlying mechanism and regulatory factors remain largely unknown [49,51,52,53]. The common denominator among evidence that supports MET-mediated secondary tumor formation is the re-expression of E-cadherin in migrating tumor cells [49,53]. The expression of E-cadherin is reduced or lost prior to the initiation of tumor invasion and metastasis [54]. E-cadherin repression in tumor cells consequently triggers the activation of EMT induction machinery that promote invasive and migratory phenotypes [54]. Hypermethylation of the E-cadherin promoter [55,56] and hypoxia [57] are among factors that repress the expression of E-cadherin in tumors. Transcription factors such as SNAIL, Slug, ZEB1, and ZEB2 can also repress E-cadherin expression by binding to the E-box of the E-cadherin promoter [19]. Future studies are required to elucidate the mechanism(s) underlying the regulation of E-cadherin expression by WAT or isolated adipocytes.

Bone marrow is one of the main sites of breast cancer metastasis [58] and is replete with marrow adipose tissues that share some biological similarities with WAT [59]. WAT in bone marrow is located in both the endosteal niche and the vascular niche with each having different gene expression, function, and ECM composition [60]. The evidence to date suggests that vascular WAT ECM is more similar to visceral WAT, while endosteal WAT ECM is more similar to subcutaneous WAT [60]. The differences in mammary WAT compared to these other depots, in patients without BC, has not been reported. While our evidence suggests that the extramedullary WAT depots from obese and lean mice have a similar ability to induce MET, further work is needed to assess if obese or lean mammary or bone marrow adipose tissue regulates MET in a similar manner.

One of the main goals of cancer therapeutics is to inhibit the metastatic dissemination of cancer cells to distal organs, since this is the major cause of mortality among patients [20]. Targeting key processes in the tumor metastasis cascade such as EMT/MET represents a viable strategy for achieving this goal. In theory, targeting EMT could abrogate the migratory and invasive potential of the tumor, thereby preventing the metastatic dissemination of tumor cells from their primary origin, whereas inhibiting MET could prevent tumor colonization in secondary organs. WAT-derived pro-MET factor(s) could be a potential therapeutic target for inhibiting metastasis formation in both lean and obese TNBC patients. This therapeutic strategy could be combined with other established treatment regimens such as chemotherapy to help ameliorate the disease progression.

## 4. Materials and Methods

### 4.1. Cell Line

MDA-MB-231 cells were obtained from American Type Culture Collection (ATCC) (Burlington, ON) and maintained in complete media (Roswell Park Memorial Institute (RPMI) 1640 Medium (Life Technologies, Burlington, ON) supplemented with 10% fetal bovine serum (FBS) (Gibco—Life Technologies) and 1% penicillin streptomycin (Life Technologies)). Cells were confirmed to be mycoplasma-free using the MycoAlert™ Plus Mycoplasma Detection Kit from Lonza (Basel, Switzerland). The cells were authenticated by STR profiling by The Centre for Applied Genomics (The Hospital for Sick Children, Toronto, ON, Canada). Cells were used up to 15 passages after initial thawing.

### 4.2. Animal Care and Experimentation

All animal procedures were approved by the Institutional Animal Use and Care Committee of the Memorial University of Newfoundland (MUN) (Protocol ID: 17-02-SC, Date: 1 September 2017). Animal handling and experimentations were carried out in strict accordance with the guidance and recommendations of the Canadian Council on Animal Care.

Five-week-old female C57/BL6 mice were obtained from Charles River Laboratories (Senneville, QC, Canada). Mice were housed in the animal care facility at MUN under ambient conditions of temperature, relative humidity, and 12 h light/dark cycle. Mice had access to standard rodent pellet diet and water *ad libitum* for a one-week acclimatization period. After this time, mice were randomly paired and stratified into two groups: one group was placed on a low-fat diet (LFD) (10% fat by kCal, D12450J, with matching sucrose to D12492; Research Diets, Inc., New Brunswick, NJ, USA) and the other group was placed on a high-fat diet (HFD) (60% fat by kCal, D12492; Research Diets, Inc., New Brunswick, NJ, USA). Mice were maintained on these diets for 10 weeks, and their body weight was measured weekly. At week 10, the body weight and fat pad weight of mice on the HFD was significantly higher than that on the LFD (Figure S1). On the 10th week, mice were anesthetized using isoflurane and sacrificed by cervical dislocation. Peri-uterine (visceral) and inguinal (subcutaneous) white adipose tissue (WAT) were isolated, weighed, and placed in transport buffer (1x phosphate-buffered saline [PBS] (137 mM NaCl, 2.7 mM KCl, 10 mM Na2HPO4, 1.8 mM KH2PO4, and pH 7.4), 5.5 mM glucose, 1% penicillin-streptomycin) at room temperature. The specimens were transported immediately to the biosafety cabinet for further processing.

### 4.3. Organotypic Culture of White Adipose Tissue

**Figure 8:**
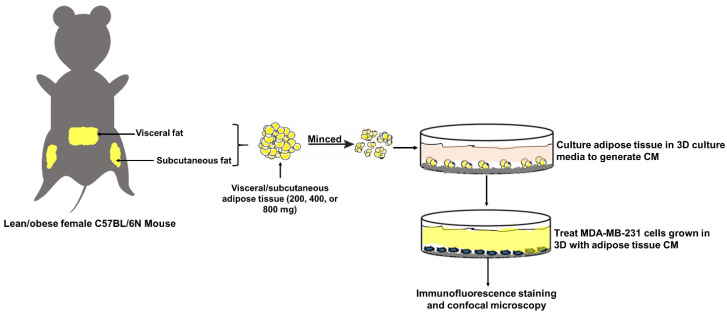
Schematic representation of the experimental design. Lean or obese female C57BL/6 mice were euthanized and 200, 400, or 800 mg of subcutaneous and visceral WAT were collected and minced into smaller tissue fragments. The minced tissue fragments were embedded in Matrigel and cultured ex vivo to generate WAT-CM. Then, MDA-MB-231 cells were culture in 3D with or without WAT-CM for five days. On the fifth day, cells were fixed with paraformaldehyde and stained for immunofluorescence confocal microscopy.

Peri-uterine and inguinal WAT (200 mg, 400 mg, or 800 mg) were washed briefly in a sterile dish with sterile PBS containing 1% penicillin streptomycin (Figure 8). Tissue samples were minced into smaller fragment using a sterile razor and washed several times with PBS over a 70 µM nylon mesh. Then, 80 µL of Matrigel (basement membrane matrix; growth factor reduced, phenol red free, Cat# 356,231, 9.1 mg/mL, BD Bioscience), which was cooled on ice, was pipetted into a 24-well plate and incubated at 37 °C for 30 min. Then, the tissue fragments were embedded in the semi-solid Matrigel and incubated for an additional 15 min at 37 °C. This procedure was used to prevent the tissue fragments from floating during culture and provide additional laminin-rich ECM. At this time, 1 mL of mammary epithelial basal media (Promo Cell, Heidelberg, Germany) containing epidermal growth factor (10 ng/mL), insulin (5 μg/mL), hydrocortisone (0.5 μg/mL), and bovine pituitary extract (0.4%) (3D culture medium) was added to the wells and incubated at 37 °C and 5% CO_2_. The WAT explants were cultured for 48 h, after which time conditioned media (CM) was collected. Fresh media was added to the WAT explants, after which they were cultured for an additional 48 h.

### 4.4. Three-Dimensional (3D) Culture

The 3D culture was performed as previously described with slight modifications [31]. Briefly, 70 µL of Matrigel was placed in an 8-well culture slide (Cat# 354,118, Corning-Life Science, New York, NY, USA) and incubated for 1 h at 37 °C to allow it to solidify. CM from WAT were mixed with fresh 3D culture medium containing 2% Matrigel in a ratio of 1:1. Then, MDA-MB-231 cells (6.750 cells total) in 500 µL of the diluted CM or 3D culture medium were overlaid on the solidified Matrigel in the chambered slide. MDA-MB-231 cells were grown for 48 h to allow colony formation. Diluted CM and 3D culture medium were replaced/replenished after 48 h, and the cells were fixed on the 5th day with 4% paraformaldehyde for 20 min at room temperature.

### 4.5. Immunofluorescence (IF) Staining

Following fixation, cells were permeabilized with PBS containing 0.5% Triton X-100 for 10 min at 4 °C, then washed 3 times with PBS-glycine (100 mM glycine in 1X PBS) and blocked with 10% donkey serum (Cat# D9663, Sigma-Aldrich, St. Louis, MO, USA) in IF buffer (0.1% BSA, 0.2% Triton X-100, 0.05% Tween 20, PBS) (blocking buffer) for 1 h at room temperature. Primary antibodies were diluted, as detailed below, in blocking buffer, incubated with cells overnight at 4 °C followed by detection with appropriate secondary antibody. Before and after secondary staining, cells were washed 3 times with 300 µL of IF buffer. Then, cells were stained with DAPI (Life technologies Carlsbad, CA, USA) for 15 min at room temperature and washed one time with 1X PBS. The chambers were gently separated from the slide according to the manufacturer’s protocol, and excess PBS was gently wiped off with a Kimwipe. An even bead of silicone sealant (GE, Boston, MA, USA) was applied around the Matrigel layer to avoid the compression of co-cultures by coverslips. Prolong Gold (Life Technologies) was used for mounting slides, coverslips were applied, and the slides were placed in a dark at room temperature to dry overnight.

Primary antibodies and dilutions used were as follows: vimentin (1:200; Cat #V2258; Sigma-Aldrich), E-cadherin (1:200; Cat #610181; BD Biosciences, Franklin Lakes, NJ, USA), Claudin-7 (1:200; Cat #AB27487; Abcam, Cambridge, UK), CD24 (1:100; Cat #NBP1-46390; Novus Biologicals, Littleton, CO, USA), and Ki67 (1:100; Cat #M724029-1; Dako, Denmark). Secondary antibodies used were AlexaFluor-647 anti-rabbit (Cat #711-605-152; Jackson Immunoresearch laboratories) and DyLight 594 anti-mouse (NBP1-75617; Novus biologicals). The slides were imaged using the 20X objective with the Nikon A1 confocal microscope with NIS elements imaging software (Nikon Inc, Tokyo, Japan). To test for possible background staining that may occur due to non-specific binding of antibodies to endogenous Fc receptors or otherwise non-specifically, MDA-MB-231 cells were incubated with mouse IgG isotype and rabbit IgG isotype antibodies that matched the isotype of the staining antibodies to be used. This was followed by secondary antibody staining with DyLight 594 anti-mouse and AlexaFluor-647 anti-rabbit, respectively (Figure S2).

### 4.6. Image Analysis

Colony morphologies were assessed by analysis of circularity using ImageJ v.1.52a [61]. Structures present in 5 images per replicate (minimum 10 structures per replicate) were traced manually, and the circularity measurement was obtained. A circularity value of >0.7 was classified as round/mass-like, 0.7–0.2 was classified as grape-like, and <0.2 was classified as stellate as reported previously [31]. Protein expression was analyzed using ImageJ by measuring the intensity of fluorescence of the whole image. Integrated density (IntDen) was determined to capture the total fluorescence of each marker. Background was subtracted by taking measurements of selected areas where cells were absent as observed from the respective bright field images. The total fluorescence intensity of each marker was determined by normalizing the IntDen of each marker to the IntDen of DAPI and then the relative IntDen values of each marker per condition were determined relative to the control. Images were altered for brightness and contrast for easier viewing, but only unaltered images were used for quantification.

### 4.7. Determination of the E-Cadherin/Vimentin Ratio of WAT-CM Treated MDA-MB-231 Cells

Log_2_-transformed E-cadherin/vimentin ratios were calculated from the immunofluorescence data of WAT-CM treated MDA-MB-231. Cells were classified as epithelial, mesenchymal, or hybrid according to previously described parameters with some slight modifications [39,40,41]. Briefly, cells that have stellate colony morphology and an E-cadherin/vimentin ratio < 0 are classified as mesenchymal. Cells with grape-like/mass-like colony morphology and an E-cadherin/vimentin ratio > 0 are considered epithelial. Cells with mixed mesenchymal and epithelial morphologies and a dissonant E-cadherin/vimentin ratio are categorized as hybrid.

### 4.8. Statistical Analysis

Statistical analysis was performed as indicated in figure legends. Differences were considered significant at *p* < 0.05. Data were analyzed using GraphPad Prism Version 8.0.1.244 (GraphPad Software, San Diego, CA, USA).

## 5. Conclusions

In conclusion, our study revealed that lean and obese mouse WAT can cause MET-like changes in mesenchymal TNBC cells via the secretion of a pro-MET factor(s). The MET induction capacity of lean and obese mice WAT was not significantly different. However, adipose tissue from the subcutaneous and visceral depots affected MET-like changes in TNBC cells differently. The lean and obese mouse subcutaneous WAT cause a much stronger MET-like effect in MDA-MB-231 cells compared to the lean visceral WAT. However, in the obese state, the visceral WAT induces a comparable MET-like effect to the lean and obese subcutaneous WAT. Translationally, we believe that WAT could play an important role in secondary tumor formation in distal sites such as the bone, lungs, or brain, albeit more work is needed to elucidate the underlying mechanisms, which is necessary to identify druggable targets.

## Supplementary Materials

Supplementary Materials can be found at https://www.mdpi.com/1422-0067/21/17/6439/s1.

## Abbreviations

3D	3-dimensional
BC	Breast cancer
CM	Conditioned media
DIO	Diet-induced obesity
EMT	Epithelial-to-mesenchymal transition
HFD	High-fat diet
IntDen	Integrated density
LFD	Low-fat diet
MET	Mesenchymal-to-Epithelial transition
TNBC	Triple negative breast cancer
WAT	White adipose tissue
