# Supplementary material for: Adipose Tissue from Lean and Obese Mice Induces a Mesenchymal to Epithelial Transition-Like Effect in Triple Negative Breast Cancers Cells Grown in 3-Dimensional Culture
Source: Int J Mol Sci. 2020 Sep 3;21(17):6439. doi: 10.3390/ijms21176439 (PMC7503907; doi:10.3390/ijms21176439)
Supplement: Supplementary file 1 [file ijms-21-06439-s001.pdf]

Adipose Tissue from Lean and Obese Mice Induces a Mesenchymal to Epithelial Transition-Like Effect in Triple Negative Breast Cancers Cells Grown in 3-Dimensional Culture

Emmanuel C. Asante, Nikitha K. Pallegar, Alica J. Hoffman Alicia M. Viloria-Petit and Sherri L. Christian

Supplementary figures S1-S2

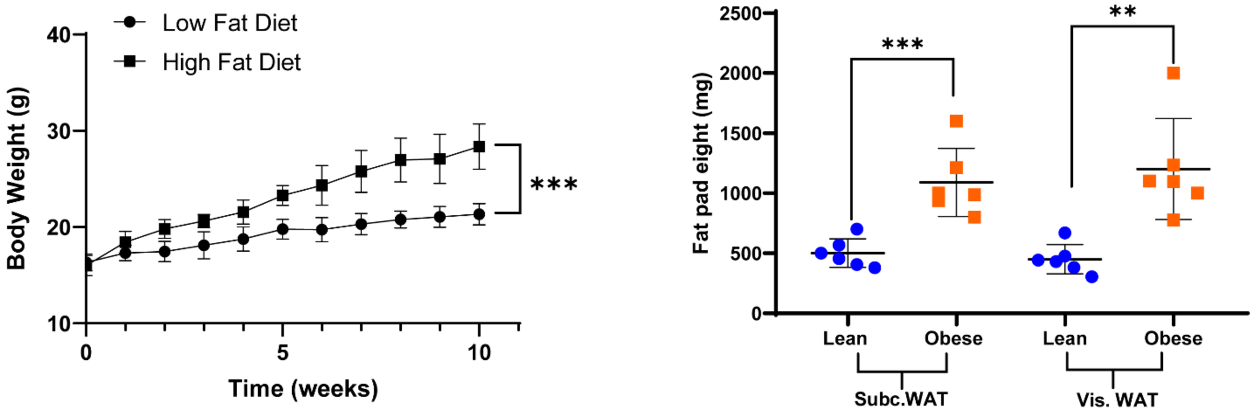

(a) (b)

Figure 1. (a) Female C57BL/6N mice becomes obese after 10 weeks on high fat diet. Body weight of C57BL/6N mice fed low fat diet or high fat diet for 10 weeks. Values are means ± SD. n = 6. Differences between means were determined by repeated measure one-way ANOVA., ***$p < 0.001$. **(b)** Fat pad weight of lean and obese female C57BL/6N mice post-euthanization. Data points are mean ± SD weight of the fat pads. n = 6. Differences between means were determined by Student's T-test, ***p <0.001 and **p <0.01.

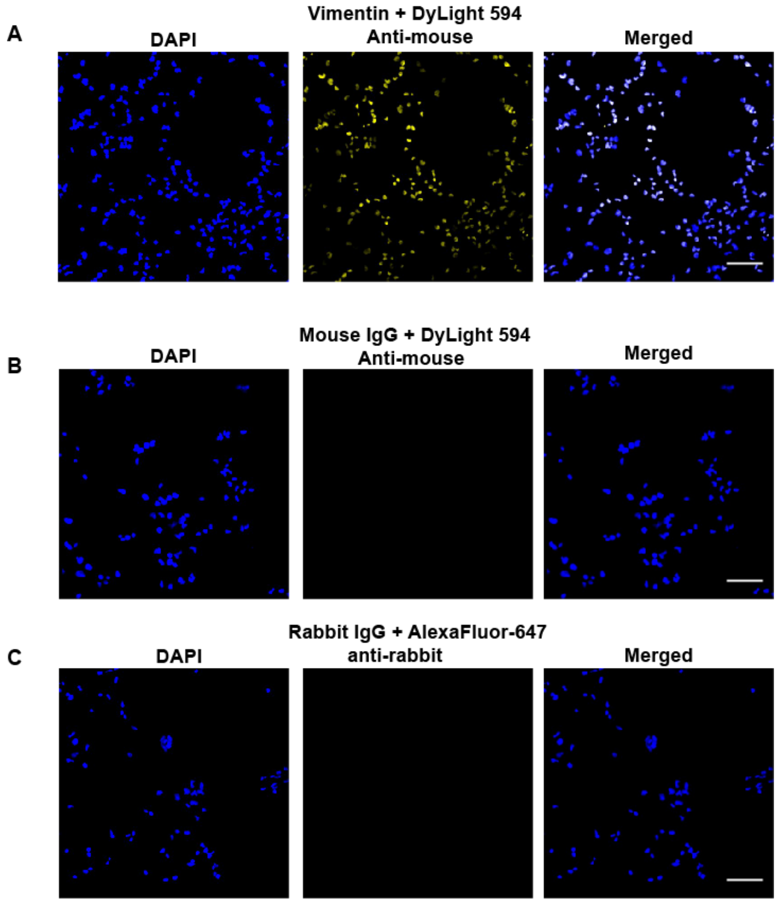

Figure 2. Signal due to non-specific antibody binding was not detected. Representative images of MDA-MB-231 cells stained with: **(A)** anti-vimentin followed by Dylight 594 anti-mouse. **(B)** mouse IgG isotype control followed by Dylight 594 anti-mouse. **(C)** rabbit IgG isotype control followed by Alexafluor 674 anti-rabbit (scale bar = 100 μM).
